# Comparative Analysis of Physical Activity Detected *via* an External Accelerometer and Cardiac Implantable Electronic Devices

**DOI:** 10.3389/fcvm.2022.898086

**Published:** 2022-05-27

**Authors:** Chun-Kai Chen, Li-Ying Cheng, Shan-Wei Hsu, Min-Tsun Liao, Po-Wen Ku, Yen-Bin Liu

**Affiliations:** ^1^Division of Cardiology, Department of Internal Medicine, National Taiwan University Hospital Hsin-Chu Branch, Hsinchu, Taiwan; ^2^School of Nursing, National Taipei University of Nursing and Health Sciences, Taipei, Taiwan; ^3^Department of Internal Medicine, College of Medicine, National Taiwan University, Taipei, Taiwan; ^4^Graduate Institute of Sports and Health Management, National Chung Hsing University, Taichung, Taiwan; ^5^Division of Cardiology, Department of Internal Medicine, National Taiwan University Hospital, Taipei, Taiwan

**Keywords:** cardiac implantable electric device, physical activity, ActiGraph GT3X+, accelerometer, validation

## Abstract

**Background:**

Physical activity (PA) has become an important health issue for decades. Cardiovascular implantable electronic devices (CIEDs) have built-in PA-recording functions. We aimed to compare PA measurements using an external accelerometer (ActiGraph GT3X+) and internal accelerometers (Abbott, Biotronik, and Medtronic CIEDs).

**Methods:**

This was a prospective, single-center observational study. The device-measured 7-day average PA was collected, and GT3X+ -measured 7-day average PA was used as the gold-standard, including all daily observations of activity. Pearson’s correlation coefficients were used to compare the correlations between GT3X+ -measured and CIED-measured PA. Bland-Altman plots were used to analyze measurement agreement, and intraclass correlation coefficients were used to analyze reliability.

**Results:**

In total, 720 patients treated with CIEDs were surveyed between November 2020 and April 2021, 60 of them were analyzed after patient screening by our protocol. Each manufacturer included 20 patients for the final analysis. The CIED-measured PAs of Abbott, Biotronik, and Medtronic were 3.0 ± 1.5, 2.6 ± 1.8, and 3.8 ± 2.5 h per day, respectively; the GT3X+ -measured PAs were 6.9 ± 2.8, 6.0 ± 2.4, and 6.4 ± 2.5 h per day, respectively. Moderate and significant correlations were found in patients using Abbott, Biotronik, and Medtronic CIEDs (*r* = 0.534, *p* = 0.015; *r* = 0.465, *p* = 0.039; *r* = 0.677, *p* = 0.001, respectively). Bland-Altman plots and intraclass correlation coefficients both showed a significant correlation and reliability between the average PA measured by GT3X+ and CIEDs (hours per day).

**Conclusion:**

Although the PA recording function of CIEDs includes a single-axis accelerometer, it has a moderate correlation compared with the triaxial accelerometer of the GT3X+. However, CIEDs seem to underestimate PA for 3–4 h compared to the GT3X+.

## Introduction

Physical activity (PA) has become an important health issue for decades. Continuous exercise has many benefits, including increased cardiopulmonary function, and reduced cardiovascular events, cardiovascular mortality, and all-cause mortality (1, 2). In older patients, exercise can promote health maintenance and improve the quality of life (3). Most patients with cardiovascular implantable electronic devices (CIEDs) are older, with an average age of approximately 75 years (4). Previous studies have shown that a decrease in PA is associated with the development of atrial fibrillation and atrial high-rate episodes, detectable by CIEDs (5, 6).

CIEDs have built-in accelerometers with a continuous and uninterrupted PA recording function, which can be used to objectively assess the PA of patients. A previous study demonstrated that patients with a basic activity of < 1 h per day had a 7.4-fold increased risk of death compared with patients who exceeded 3 h per day (7), while another study demonstrated that a decrease in PA significantly increased the risk of hospitalization for acute decompensated heart failure within the subsequent 30 days (8). Although accelerometers in CIEDs can continuously record the amount of PA in patients over a long period, there are still differences regarding the detection threshold and processing algorithm between various manufacturers. Important activity sensor thresholds and signal processing algorithms have not been fully elucidated.

GT3X+ (ActiGraph, Pensacola, FL, United States) is popular in research and is the gold standard tool for measuring PA (9, 10). Therefore, the purpose of this study was to investigate the differences in PA levels measured using Abbott, Biotronik, and Medtronic CIEDs, compared with the external accelerometer of the ActiGraph GT3X+.

## Materials and Methods

### Study Design and Participants

**FIGURE 1:**
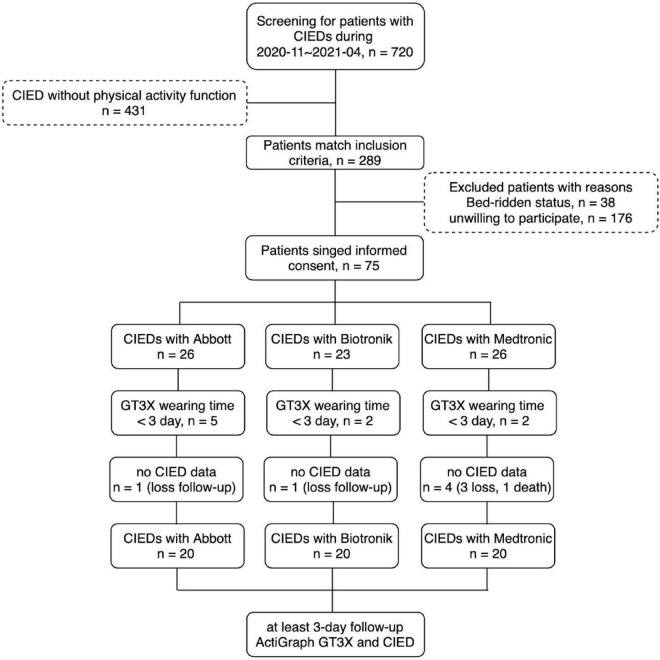
Study flow diagram.

This was a prospective, single-center, longitudinal observational study; all enrolled patients were followed-up for 7 days. This study was approved by the institutional review board of the National Taiwan University Hospital Hsin-Chu Branch, and all participants provided written informed consent. The flow diagram of the study design is shown in Figure 1. Patients with CIEDs were consecutively enrolled at the National Taiwan University Hospital, Hsin-Chu Branch, Taiwan. The inclusion criteria were: an age of 20–100 years; use of a CIED with PA measurement function; and CIED implantation for more than 30 days without complications. The exclusion criteria were: patients or legal representatives who could not provide informed consent; being unwilling or unable to return for follow-up visits, or reason to believe that adherence to follow-up visits would be irregular; current or scheduled enrollment in other conflicting studies; concomitant disease (e.g., terminal cancer) or other medical conditions likely to result in death within 6 months; bedridden patients who could not ambulate; and pregnancy.

If possible candidates were admitted to the hospital or visited the outpatient department, a specially trained nurse screened the electronic medical records and assessed their eligibility according to the inclusion and exclusion criteria of the study physicians. Patients were verbally informed of the study and signed a consent form. All patients underwent history-taking and physical examinations, and the 7-day average PA was measured using devices from each manufacturer. Patients wore the GT3X+ on their wrists with 30 hertz sampling rate for 7 days, after which they returned the device to the hospital. The activities were all recorded as long as the GT3X+ wore on their wrist. If the GT3X+ was not worn by the patient, it would label non-use as sedentary or sleeping time. The 7-day average PA was defined as the average hours per day recorded either by the CIED or GT3X+ in the study period.

### ActiGraph GT3X+ -Measured Physical Activity

The GT3X+ is a triaxial external accelerometer capable of detecting three planes of body motion that provide physiological activity measurements. Mainly, it includes daily activity count [vector magnitude units (VMUs)], x-, y-, and z-axis counts, vector amplitude, energy consumption, pedometer (steps per day), activity intensity level—including total hours of light, moderate, vigorous, and very vigorous PA—sedentary time, and metabolic equivalent. The GT3X+ is equipped with an inclinometer to judge the posture of patients, as well as determine when the device is removed. The reliability of internal and external measurements is high, and energy consumption, although measured indirectly, is highly correlated with the actual energy consumption (11).

### Device-Measured Physical Activity

CIED accelerometers were originally designed for rate-adaptive pacing (12). When detecting patient activity, rapid cardiac stimulation is performed to achieve a faster heart rate. This function is consistent with the original physiological changes that occur in the human body during activity, as a rapid heart rate increases the cardiac output and provides more energy to the cells of the body. Therefore, CIED accelerometers are single-axis detectors that may have more restrictions on motion monitoring compared to triaxial external accelerometers. However, there are different detection and recording algorithm for PA among different manufacturer. For example, when the accelerometer embedded in the Abbott (Abbott Laboratories, Chicago, IL, United States) CIED detects body motion for more than 1 min, it starts recording the activity time; when body motion is not detected for more than 2 min, the recording is stopped (13). In contrast, the PA detected using the Biotronik (Berlin, Germany) CIED was delivered at a rate exceeding the resting heart rate (14). The activity sensor threshold of the Medtronic (Dublin, Ireland) CIED is equivalent to an active minute of approximately 70 steps per minute (15). Abbott and Medtronic CIEDs report daily PA as hours per day (13, 16), while daily PA detection by Biotronik is based on the percentage of time per day (14). Nevertheless, CIED accelerometers are still advantageous owing to their long-term, continuous, and uninterrupted PA monitoring.

The CIED data were wirelessly extracted through an interrogation machine using the software of each manufacturer. All PA records were recorded, thus providing a long-term PA trend. In this study, the raw data of various manufacturers could not be obtained; therefore, we used the PA trend graph to estimate the 7-day CIED-measured average PA. The graph of the PA trend was extracted for the period of interest (Supplementary Figure 1), and ImageJ was used to calculate the percentage of the area of the graph above and below the curve (17); the actual PA value was calculated using the percentage of the area under the curve (Supplementary Figures 1A–C). The Medtronic CIED presented the average weekly PA over the previous 7 days (Supplementary Figure 1C); thus, weekly PA was used to justify the credibility of this approach.

### Outcome Measurement

The parameters of the GT3X+ included daily activity, step, and VMU observations for each subject (hours per day); x/y/z axes; 7-day average activity; sedentary, light, moderate, and vigorous activity; 7-day average steps per day; and 7-day average VMU per day. For the Abbott and Medtronic CIEDs, PA was measured in hours per day; for the Biotronik CIED, PA was measured as percentage of time per day and was converted to hours per day (20% per day equaled 4.8 h per day). The primary purpose of this study was to compare the 7-day average PA (hours per day) between the GT3X+ and each CIED-measured 7-day average PA (hours per day). The CIEDs of the three manufacturers were then compared with the gold standard method (ActiGraph GT3X+). For secondary purposes, the 7-day average PAs (hours per day) measured using CIEDs were compared with the 7-day average steps per day, 7-day average VMUs, and x/y/z axes measured using the GT3X+.

### Statistical Analysis

Continuous variables were presented as mean ± standard deviation (*SD*) or median (interquartile range); categorical variables were reported as numbers (percentages). One-way analysis of variance or the Kruskal-Wallis test was used to compare continuous data between the three manufacturers (Abbott, Biotronik, and Medtronic), and categorical data were compared using the Chi-squared test. Pearson correlation coefficients were used to compare correlations between the 7-day average PA including total, light, moderate, and vigorous activity using the GT3X+, and the CIEDs from the three manufacturers (Abbott, Biotronik, and Medtronic). Scatter plots were used to compare the 7-day average activity (hours per day) between the CIEDs and GT3X+. In addition, comparison of correlations from independent three groups were also analyzed by transforming the correlation coefficient value into z scores. Bland-Altman plots were used to analyze the measurement agreement between the GT3X+ and each manufacturer using the average PAs of each device (18). Intraclass correlation coefficients (ICCs) were also used to analyze the average PA measured by the GT3X+ and each manufacturer.

The required sample size was calculated using the Bland-Altman plot *via* the method described by Lu et al. (19), while the *a priori* sample size was estimated based on a previous study (20); the mean difference was 0.77 ± 0.99 h per day. The maximum allowed difference between the GT3X+ and CIEDs was 4.04 h per day. When controlling for baseline values with 85% power and a type I error of 0.05, the sample size was 20 participants for each manufacturer. Allowing for a 20% dropout rate, we aimed to enroll 25 patients from each manufacturer; thus, 75 prespecified participants were enrolled in this study. Significant differences between groups were reported at an alpha level of 0.05, and all reported *P*-values were two-sided. Statistical analyses were performed using SPSS version 22.0 for Windows (SPSS Inc., Chicago, IL, United States).

## Results

### Outcomes

In total, 720 patients with CIEDs were surveyed between November 2020 and April 2021. The CIEDs of 431 patients did not have a PA recording function; thus, 289 patients were eligible for this study. Thirty-eight patients were bedridden, and 176 patients were unwilling to participate in the study; this was mainly because they were unwilling to return to the clinic after 7 days, or were inconvenient to wear the GT3X+.

In total, 75 patients received the ActiGraph GT3X+. Among them, nine patients had a wearing time of less than 3 days, and six patients had no follow-up data for CIED-measured PA (5 were lost to follow-up and 1 died). The CIEDs from the three manufacturers included 20 different patients in the final analysis (Figure 1). The basic characteristics of the three patient groups are shown in Table 1; there were no significant differences regarding basic characteristics, including age, sex, various diseases, and medications.

**TABLE 1:** Baseline characteristics of study participants.

Clinical characteristics	Abbott *N* = 20	Biotronik *N* = 20	Medtronic *N* = 20	*p*-value
**Demographic and medical history**				
Age (year)	77.5 ± 12.7	74.6 ± 12	70.4 ± 18.5	0.312
Male	9 (45)	10 (50)	8 (40)	0.817
BMI	25.1 ± 3.3	24.7 ± 3.8	25 ± 5.9	0.955
Indication for PPM				0.344
SSS	15 (75)	17 (85)	13 (65)	
AVB	5 (25)	3 (15)	7 (35)	
Hypertension	10 (50)	14 (70)	11 (55)	0.410
Diabetes mellitus	6 (30)	6 (30)	6 (30)	1.000
Coronary artery disease	4 (20)	4 (20)	1 (5)	0.308
Congestive heart failure	3 (15)	2 (10)	2 (10)	0.851
Cerebral vascular accidence	2 (10)	2 (10)	1 (5)	0.804
Atrial fibrillation	3 (15)	4 (20)	3 (15)	0.887
Peripheral artery disease	0 (0)	0 (0)	1 (5)	0.362
CKD	1 (5)	2 (10)	2 (10)	0.804
ESRD	1 (5)	0 (0)	1 (5)	0.596
COPD	2 (10)	2 (10)	0 (0)	0.343
Hyperlipidemia	8 (40)	4 (20)	5 (25)	0.344
Smoke	5 (25)	8 (40)	4 (20)	0.344
NYHA status				
No heart failure	17 (85)	18 (90)	18 (90)	0.838
Class I	2 (10)	1 (5)	2 (10)	
Class II	1 (5)	1 (5)	0	
Class III	0	0	0	
**Pharmacologic therapy**				
Alpha-blockers	0 (0)	3 (15)	1 (5)	0.153
Beta-blockers	7 (35)	5 (25)	5 (25)	0.720
ACEi/ARB	8 (40)	6 (30)	2 (10)	0.092
Calcium channel blockers	7 (35)	7 (35)	8 (40)	0.931
Diuretics	6 (30)	3 (15)	5 (25)	0.521
Antiarrhythmia agents	1 (5)	1 (5)	1 (5)	1.000

*BMI, body mass index; NYHA, New York Heart Association; LVEF, left ventricular ejection fraction; ACEi, angiotensin-converting-enzyme inhibitor; ARB, angiotensin receptor blocker.*

*Continuous values are expressed as mean ± standard deviation (SD) or median (interquartile range); categorical variables are reported as numbers (percentages).*

### ActiGraph GT3X+ -Measured Physical Activity

Most of the study subjects completed a near 7-day wearing time of GT3X+, and there was no statistical difference of wearing times between groups (Abbott 6.7 ± 0.56 days, Biotronik 6.55 ± 0.75 days, Medtronic 6.96 ± 0.08 days, *p* = 0.065). Among the data measured by the GT3X+, there were no statistically significant differences regarding the 7-day average daily activity, or daily light, moderate, or vigorous activity. Regarding very vigorous activity measured by the GT3X+, there were no available measurements, indicating that very vigorous activity was not performed by the study patients. Additionally, there were no significant differences between the three groups regarding 7-day average steps per day, 7-day average VMU per day, y-axis per hour (vertical), x-axis per hour (anterior-posterior), z-axis per hour (medial-lateral), average kcal per day, and metabolic equivalent of task (MET) (Table 2).

**TABLE 2:** ActiGraph GT3X+ and cardiovascular implantable electronic device-measured physical activity of study participants.

GT3X+ parameters	Abbott *N* = 20	Biotronik *N* = 20	Medtronic *N* = 20	*p*-value
Device-measured PA (hours per day)	3.0 ± 1.5	2.6 ± 1.8	3.8 ± 2.5	0.126
Wearing days of ActiGraph	6.7 ± 0.56	6.55 ± 0.75	6.96 ± 0.08	0.065
7-day average activity (hours per day)	6.9 ± 2.8	6.0 ± 2.4	6.4 ± 2.5	0.531
Light activity (hours per day)	4.3 ± 1.6	3.9 ± 1.2	3.9 ± 1.1	0.487
Moderate activity (hours per day)	2.4 ± 1.4	2 ± 1.3	2.2 ± 1.4	0.678
Vigorous activity (hours per day)	0.2 ± 0.3	0.2 ± 0.2	0.3 ± 0.4	0.459
7-day average steps per day	8,276 ± 4,769	6,949 ± 4,486	7,537 ± 4,515	0.660
7-day average VMU per day	1,487,187 ± 816,746	1,269,637 ± 765,616	1,449,361 ± 902,263	0.677
y (vertical) axis per hour	33,732 ± 18,858	27,438 ± 16,623	32,742 ± 20,955	0.530
x (anterior-posterior) axis per hour	33,530 ± 19,305	28,036 ± 16,357	33,577 ± 21,172	0.575
z (medial-lateral) axis per hour	39,368 ± 21,426	35,285 ± 22,119	37,853 ± 23,268	0.843
Average kcal per day	68.9 ± 39	55.9 ± 36.9	61.5 ± 39.8	0.565
MET	1.4 ± 0.3	1.3 ± 0.2	1.4 ± 0.3	0.467

*MET, metabolic equivalent; PA, physical activity; VMU, vector magnitude units.*

### Correlation Between Cardiovascular Implantable Electronic Devices- vs. GT3X+ -Measured Physical Activity

The most important measured PAs of the Abbott, Biotronik, and Medtronic CIEDs in this study were 3.0 ± 1.5 h per day, 2.6 ± 1.8 h per day, and 3.8 ± 2.5 h per day, respectively (Table 2). Other important parameters were all activities measured by the GT3X+, including light, moderate, and vigorous activity; these were 6.9 ± 2.8 h per day, 6.0 ± 2.4 h per day, and 6.4 ± 2.5 h per day for the Abbott, Biotronik, and Medtronic devices, respectively (Table 2). The correlation comparison showed that the PA measured by the Medtronic device significantly correlated with various data measured by the GT3X+, including 7-day average activity (*r* = 0.677, *p* = 0.001), light activity (*r* = 0.457, *p* = 0.043), moderate activity (*r* = 0.696, *p* = 0.001), vigorous activity (*r* = 0.655, *p* = 0.002), daily steps (*r* = 0.666, *p* = 0.001), daily vector magnitude units, x/y/z axes (*r* = 0.651, *p* = 0.002; *r* = 0.730, *p* < 0.001; *r* = 0.687, *p* = 0.001, respectively), daily calories (*r* = 0.651, *p* = 0.002), and MET (*r* = 0.694, *p* = 0.001). The activity part is the most relevant to moderate activity, and the axis part is the most relevant to the y-axis (vertical). PA measured using the Abbott device only significantly correlated with the 7-day average PA and light activity of the GT3X+ (*r* = 0.534, *p* = 0.015; *r* = 0.559, *p* = 0.010). For the Biotronik device, light PA and daily steps measured by the GT3X+ had no significant correlation; other data were significantly correlated, with the most relevant dominated by vigorous activity; the axis part is the most relevant for the z-axis (medial-lateral) (Table 3).

**TABLE 3:** Correlations between cardiovascular implantable electronic device-measured physical activity and Actigraph GT3X+ parameters.

GT3X+ parameters	Abbott *r*	*p*-value	Biotronik *r*	*p*-value	Medtronic *r*	*p*-value
7-day average activity (hours per day)	0.534	0.015	0.465	0.039	0.677	0.001
Light activity (hours per day)	0.559	0.010	0.336	0.147	0.457	0.043
Moderate activity (hours per day)	0.413	0.070	0.445	0.049	0.696	0.001
Vigorous activity (hours per day)	0.248	0.292	0.475	0.035	0.655	0.002
7-day average steps per day	0.398	0.082	0.352	0.128	0.666	0.001
7-day average VMU per day	0.419	0.066	0.583	0.007	0.693	0.001
x(anterior-posterior) axis	0.401	0.080	0.699	0.001	0.651	0.002
y(vertical) axis	0.422	0.064	0.666	0.001	0.730	< 0.001
z(medial-lateral) axis	0.416	0.068	0.726	< 0.001	0.687	0.001
Average kcal per day	0.385	0.093	0.662	0.001	0.651	0.002
MET	0.364	0.115	0.614	0.004	0.694	0.001

*VMU, vector magnitude units.*

**FIGURE 2:**
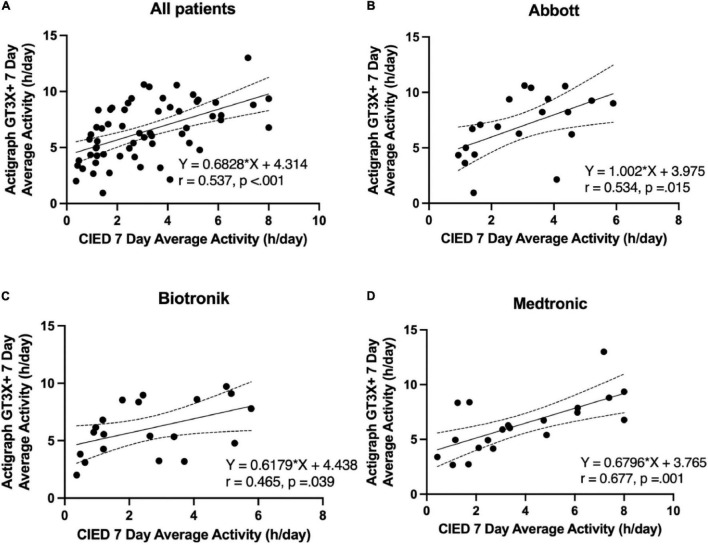
Correlation between the 7-day average physical activity (hours per day) of the pacemakers and ActiGraph GT3X+. **(A)** All patients, **(B)** Abbott, **(C)** Biotronik, **(D)** Medtronic.

CIED-measured PAs demonstrated a moderate and significant correlation with GT3X+ -measured PA in all patients (*r* = 0.537; *p* < 0.001; GT3X+ -PA = 0.6828*CIED-PA + 4.314; Figure 2A). Moderate and significant correlations were found in patients treated with Abbott (*r* = 0.534; *p* = 0.015; GT3X+ -PA = 1.002 × CIED-PA + 3.975; Figure 2B), Biotronik (*r* = 0.465, *p* = 0.039; GT3X+ -PA = 0.6179 × CIED-PA + 4.438; Figure 2C), and Medtronic CIED (*r* = 0.677; *p* = 0.001; GT3X+ -PA = 0.6796 × CIED-PA + 3.765; Figure 2D). Comparisons of correlations between CIED-measured and GT3X+ -measured average daily PA from independent three groups demonstrated no significant differences (Abbott *r* = 0.534 vs. Biotronik *r* = 0.465, *z* = 0.268, *p* = 0.394; Abbott *r* = 0.534 vs. Medtronic *r* = 0.677, *z* = −0.665, *p* = 0.253; Biotronik *r* = 0.465 vs. Medtronic *r* = 0.677, *z* = −0.933, *p* = 0.176).

**FIGURE 3:**
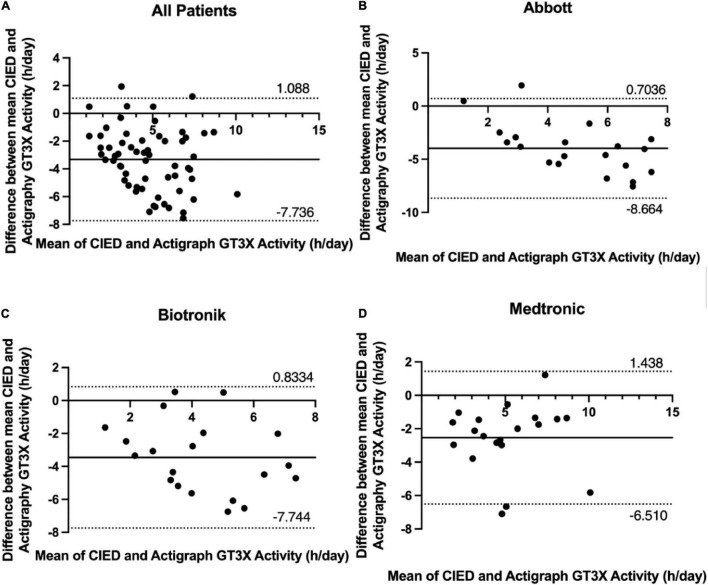
Bland-Altman difference plot of device- and ActiGraph-measured 7-day average physical activity (hours per day) with 95% limits of agreement (± 1.96 SD). **(A)** All patients, **(B)** Abbott, **(C)** Biotronik, **(D)** Medtronic.

A Bland-Altman plot including all patients (displaying differences in daily GT3X+ and CIED measurements against their means) revealed symmetrical daily activity (95% limits of agreement considering multiple observations in the same subjects, −7.736–1.088 h; Figure 3A). Bland-Altman plots for patients using Abbott, Biotronik, and Medtronic devices revealed symmetrical daily activities (95% limits of agreement −8.664–0.7036 h, −7.744–0.8334, and −6.510–1.438 h, respectively; Figures 3B–D).

The ICCs between the average PA measured by the GT3X+ and CIEDs (hours per day) were 0.614 (95% confidence interval [CI]: 0.026, 0.847; *p* = 0.022), 0.618 (95% CI: 0.034, 0.849; *p* = 0.021), and 0.807 (95% CI: 0.513, 0.924; *p* < 0.001) for the Abbott, Biotronik, and Medtronic devices, respectively (Table 4).

**TABLE 4:** Intraclass correlation coefficient between average daily physical activity measured by the Actigraph GT3X+ and cardiovascular implantable electronic devices (hours per day).

	ICC (95% CI)	F test with true value 0			
		Value	df1	df2	*p*-value
Abbott	0.614 (0.026, 0.847)	2.593	19	19	0.022
Biotronik	0.618 (0.034, 0.849)	2.615	19	19	0.021
Medtronic	0.807 (0.513, 0.924)	5.184	19	19	0.000

## Discussion

This is the first study to compare PA monitoring using accelerometers from different CIED manufacturers, with the gold standard triaxial accelerometer (ActiGraph GT3X+) serving as the benchmark. The main findings of this study are as follows: (1) the CIED accelerometers used to measure and monitor PA function had moderate correlations (21), *r* = 0.465–0.677 and moderate to good reliabilities (22), ICCs = 0.614–0.807 with the gold standard triaxial accelerometer (ActiGraph GT3X+); (2) the PA detected using a CIED accelerometer was significantly lower than detected using the standard triaxial accelerometer (ActiGraph GT3X+), with a difference of 3–4 h per day; (3) there are differences in accelerometers between manufacturers—in particular, the Medtronic devices demonstrated a better correlation (*r* = 0.677) but no statistically significant difference to Abbott and Biotronik devices; and (4) the intensification of PA measured by CIEDs may vary among manufacturers. The PA measured by Abbott devices may have been dominated by light PA; conversely, Biotronic CIEDs favored vigorous PA, while Medtronic CIEDs favored moderate PA. When detecting vigorous PA, the Abbott CIEDs were less correlated with the GT3X+, whereas the Biotronik and Medtronic CIEDs were significantly correlated. When detecting light PA, Biotronik CIEDs were less correlated, whereas Abbott and Medtronic CIEDs were significantly correlated (Supplementary Figure 2). This may be due to the different software settings and accelerometer designs of various manufacturers.

The hypothesis of this study is that PA monitoring using CIEDs highly correlates with PA monitoring using the GT3X+, and varies among manufacturers. The results of this study supported this hypothesis. Three validation studies have been conducted regarding the accelerometer function of CIEDs compared with external accelerometers (20, 23, 24). One compared the Medtronic implantable cardioverter defibrillator (ICD) and cardiac resynchronization therapy (CRT) with the GT3X+; results demonstrated that the amount of PA measured using the Medtronic CIED highly correlated with the amount of PA measured by the GT3X+ (*r* = 0.831) (20). In another study, the weekly PA measured using the Biotronik CIED, and the metabolic equivalents obtained using the GT3X+, were only low to moderately correlated (*r* = 0.37) (23). The third study compared the PA detected by the Medtronic CIED, with that detected by the external accelerometer of the ApierMotion 440. A strong individual correlation was found; however, there was a substantial difference regarding the total amount of daily PA among patients (24).

The best model in our study was also the Medtronic CIED (*r* = 0.677), indicated by the correlation coefficient. The difference between the previous study and our own was the source population; since previous studies were mainly based on ICDs or CRT, patients were relatively more fragile than in our study. The PA measured in that study (20) was lower than in our study, with light activity accounting for more than 95% of activity. Our study included moderate and vigorous activity for one-third of the total activity time; therefore, it is known that CIED-measured PA mainly includes light to moderate activity, which may be more related to light activity. In more clinically fragile patients, the use of CIEDs to measure and monitor PA levels may therefore be more beneficial than in patients with normal activity levels, as the use of CIEDs to measure and monitor activity levels may be less accurate and may underestimate the true PA in these patients.

Here, we found that CIED-measured PAs were lower than the GT3X+ -measured PA (mean, 2.6–3.8 vs. 6.0–6.9 h per day). This may be because the threshold of the CIED accelerometers was set to a higher activity for recording. If we simply look at the durations of moderate and vigorous activity—which were around 2–2.5 h per day—it can be inferred that the CIED accelerometer threshold may have been set to light-to-moderate activity (25). However, it is also possible that the accelerometer settings exceeded a certain threshold and thus did not record outliers, making it easier to record light-to-moderate activity. This phenomenon was observed in a previous study (20) demonstrating that the difference between GT3X+ and ICD/CRT activity was not large (approximately 0.8 h); however, the difference in our study was 3–4 h.

PA has been discussed as an increasingly important issue in the past decades. In the future, changes in PA could be used for both medical care and health monitoring, as a decline in PA was found to be associated with adverse cardiovascular events and death in a previous study (26). Immediately monitoring the decline in PA can thus help prevent progression to death as early as possible. Additionally, sedentary time may be more important than PA (27, 28); therefore, if CIED-measured PA is confirmed to be a feasible way to monitor physical health, we suggest that in the future, CIED accelerometers be developed to fulfill additional functions, such as recording sedentary and sleeping time. PA is particularly useful as a function of clinical monitoring, with continuous and uninterrupted monitoring records of CIED-measured PA and fast transmission to a cloud system. CIED-measured PA may therefore play a significant role in smart medical care.

This study had some limitations. First, as the actual PA levels of the manufacturers could not be obtained, the data analyzed were extracted from the data provided by the graph; thus, there may be slight differences. However, this difference was corrected by the actual 7-day data of the Medtronic device, and the difference was not large. Second, the designs of CIED did not differentiate between light, moderate and vigorous PA. The distinction between activity levels are essential for health recommendations. The design, algorithm and threshold of activity detection among different manufacturers remained a commercial confidential information. The future design of PA detections by CIED should also focus on different activity levels, sedentary time, and sleep time. Third, this study did not analyze the different activity patterns between day-time and sleeping time. Although the GT3X+ data had the PA records at day-time and sleeping time, the CIED data only had the PA data as hours per day or percentage of time per day. Fourth, the GT3X+ does not record activity if not worn by the patient, thus labeling non-use as sedentary or sleeping time. The GT3X+ -measured PA may therefore be underestimated; however, as the PA in this study was much higher than measured using CIEDs, these differences may not be statistically significant. Fifth, the location of the CIED implantation was below the clavicle and under the chest, in the subcutaneous area, while the GT3X+ was worn on the wrist. A previous study indicated no significant differences in sleeping time measurement between dominant and non-dominant wrist ActiGraph (29). However, a GT3X+ worn on the hip may be superior for sleep timing and quantity metrics, whereas the wrist may be superior for sleep quality metrics (30). The amount of activity measured by CIEDs or the GT3X+ may be different. Hand movement alone was easier to detect using the GT3X+; however, we found it difficult to detect hand movement using CIED accelerometers. Still, this study demonstrated statistically significant moderate to strong correlations. Last, the sample size of this study was small; therefore, the mathematical equation obtained may not be accurate. Whether this finding can be extrapolated to other groups, such as patients using ICDs or CRT, remains to be investigated. Nevertheless, this study identified the most important moderate-to-high correlations, providing strong evidence for the clinical use of CIED-measured PA for intelligent healthcare through continuous and remote monitoring functions.

## Conclusion

Although the PA recording function of CIEDs includes a single-axis accelerometer, it had a moderate correlation compared with the triaxial accelerometer of the gold standard GT3X+. Additionally, while there may be differences in accelerometer function between manufacturers, CIEDs seem to underestimate PA for 3–4 h compared to the GT3X+.

## Data Availability Statement

The original contributions presented in the study are included in the article/Supplementary Material, further inquiries can be directed to the corresponding author/s.

## Ethics Statement

The studies involving human participants were reviewed and approved by the Institutional Review Board of the National Taiwan University Hospital Hsin-Chu Branch. The patients/participants provided their written informed consent to participate in this study.

## Author Contributions

S-WH, C-KC, M-TL, and Y-BL contributed to the conception and design of the study. L-YC collected the data and organized the database. P-WK managed the accelerometer data extraction and analysis. M-TL performed statistical analysis and interpretation. C-KC and M-TL wrote the manuscript. Y-BL confirmed the final approval of manuscript. All authors contributed to manuscript revision, read, and approved the submitted version.

## Conflict of Interest

M-TL received funding from Medtronic and speaker/consulting fees from Medtronic, Abbott, and Biotronik. The remaining authors declare that the research was conducted in the absence of any commercial or financial relationships that could be construed as a potential conflict of interest.

## Publisher’s Note

All claims expressed in this article are solely those of the authors and do not necessarily represent those of their affiliated organizations, or those of the publisher, the editors and the reviewers. Any product that may be evaluated in this article, or claim that may be made by its manufacturer, is not guaranteed or endorsed by the publisher.
