# Supplementary material for: Comparative Analysis of Physical Activity Detected via an External Accelerometer and Cardiac Implantable Electronic Devices
Source: Front Cardiovasc Med. 2022 May 27;9:898086. doi: 10.3389/fcvm.2022.898086 (PMC9184442; doi:10.3389/fcvm.2022.898086)

Supporting Figure 1. A representative method to demonstrate data extraction from the physical activity trend of graphs of (A) Abbott (physical activity: 2.38 h per day), (B) Biotronik (physical activity: 6.25 h per day), and (C) Medtronic (4.57 h per day) cardiovascular implantable electronic devices. The physical activity estimated using this method in the Metronic device was very close to the actual physical activity (4.6 h per day).

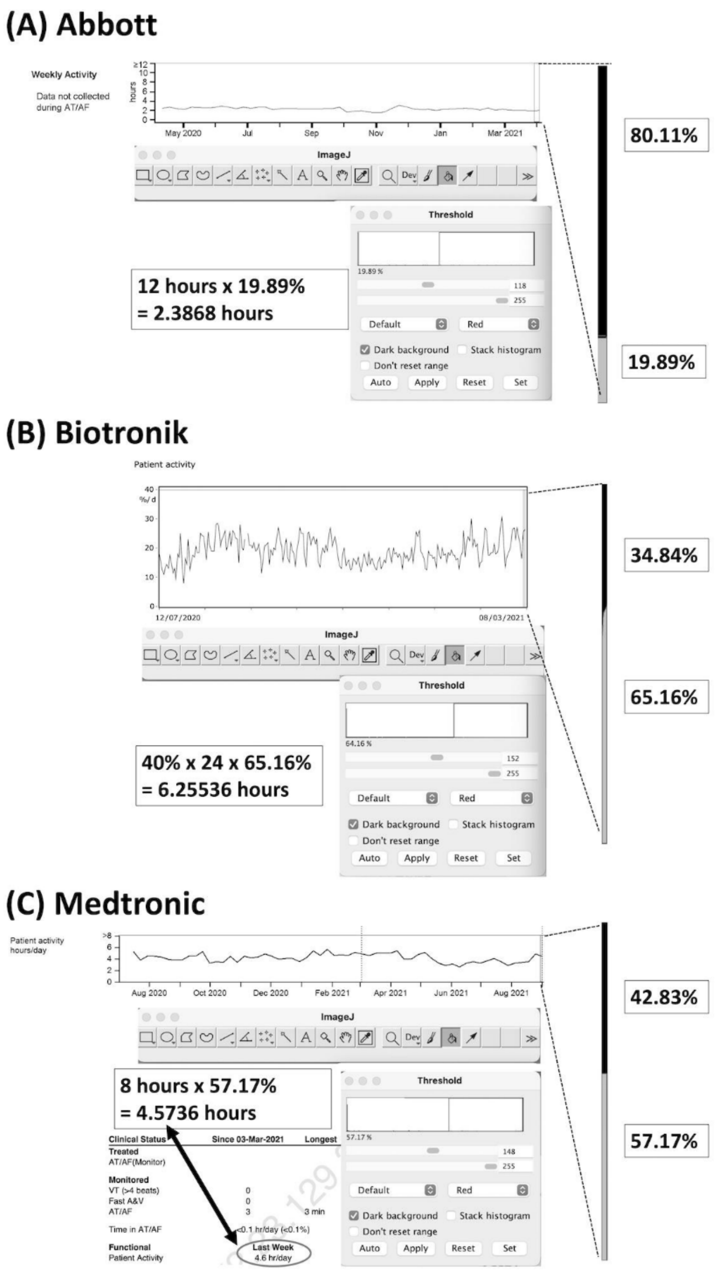

Supporting Figure 2. Correlation between the average physical activity (hours per day) of the Abbott, Biotronik, and Medtronic pacemakers (and ActiGraph GT3X+ measured light, moderate, and vigorous physical activity

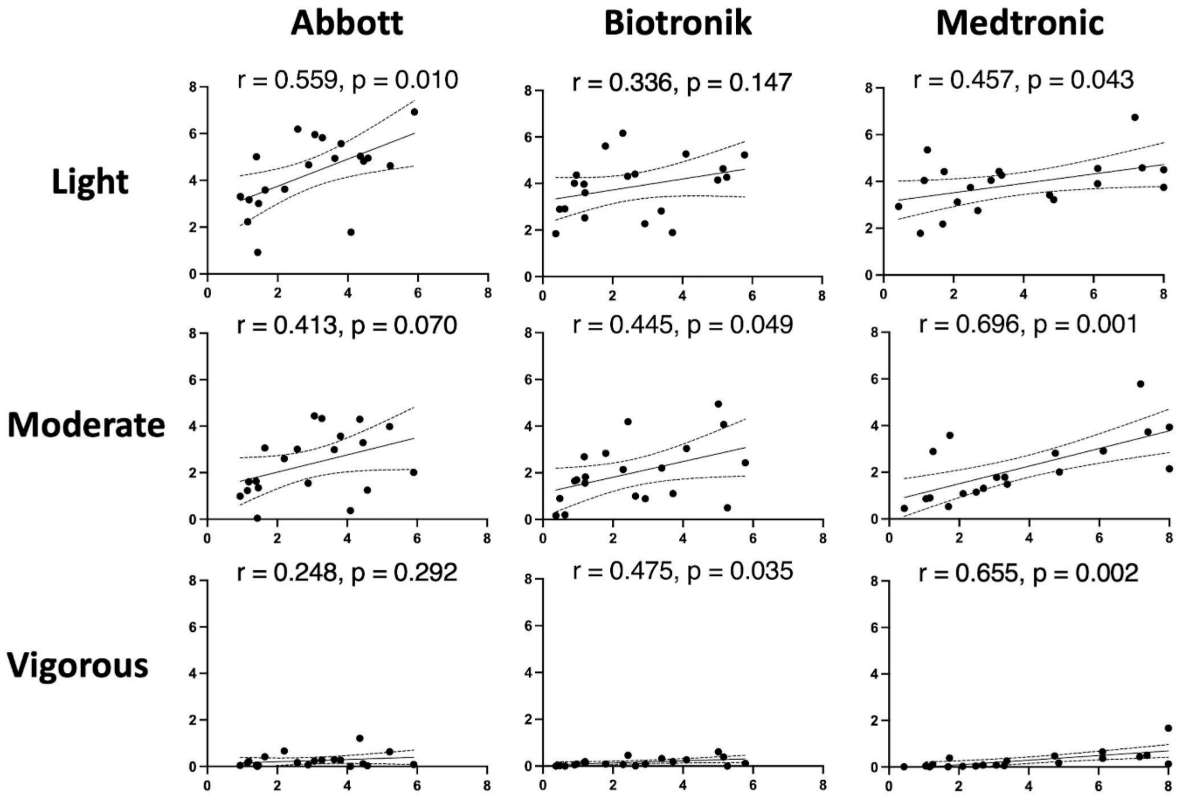

Supplement: Supplementary file 1 [file Data_Sheet_1.pdf]
